# IoTKITs: A novel dataset for IoT education kit recognition

**DOI:** 10.1016/j.dib.2025.111650

**Published:** 2025-05-12

**Authors:** Thanh-Thien Nguyen, Anh-Tuan Nguyen Do, Duc-Lung Vu

**Affiliations:** a University of Information Technology, Ho Chi Minh City, Vietnam; b Vietnam National University, Ho Chi Minh City, Vietnam; c Eastern International University, Binh Duong, Vietnam

**Keywords:** IoT Kit, Object detection, Deep Learning

## Abstract

This paper introduces IoTKITs, a novel and well-annotated dataset specifically designed for the identification and classification of IoT education kits (KITs), addressing the scarcity of publicly available datasets in this domain. The dataset comprises over 3,000 high-resolution images of various KITs, including popular designs such as Arduino Uno, Arduino Nano, ESP32, and others, with detailed annotations for object detection tasks. To establish baselines, we evaluated state-of-the-art object detection models, including YOLOv5, YOLOv7, Faster R-CNN, and SSD, on the dataset. IoTKITs is designed to advance KIT classification research and foster applications in education, embedded systems, and smart learning environments.

Specifications Table

**Table:** 

Subject	Computer Sciences
Specific subject area	Recognition and classification of IoT boards, including Arduino, Raspberry Pi, and others
Type of data	2D RGB images (.jpg, .jpeg) Annotations: JSON format (.json)
Data collection	Images were collected from multiple sources including Kaggle, Roboflow Universe, and original photos taken by the authors. All images were manually annotated using the Smart Polygon tool in Roboflow, which allows labeling objects with polygon masks instead of traditional 4-point bounding boxes.
Data source location	Various online sources (Kaggle, Roboflow Universe) and original photographs; not limited to a specific geographic location.
Data accessibility	Repository name: Mendeley Data Data identification number: 10.17632/x5thzmkxhy.1 Direct URL to data: https://data.mendeley.com/datasets/x5thzmkxhy/1
Related research article	*None*

## 1 Value of the Data

• The dataset fills a gap in publicly available image data for full IoT education kits.
• It provides annotated high-resolution images from real-world and controlled environments.
• The dataset enables reproducibility and benchmarking of CNN-based models for KIT classification in educational settings.

## 2 Background

IoT education kits (KITs) are essential components in modern electronics and STEM education, serving as platforms for learning embedded systems, programming, and hardware integration. While numerous public datasets exist for PCB defect detection and component-level recognition, they typically focus on industrial boards or surface-level analysis.

For example, datasets such as ElectroCom 61 [1], DeepPCB [2], FPIC [3], and VisA [4] support tasks like identifying ICs, resistors, and defects such as cracks or missing components. However, they do not address the holistic classification of full IoT education kits, such as Arduino or Raspberry Pi, which are commonly used in classrooms and university labs.

Recent works using YOLO-based models and hybrid data inputs (e.g., RGB + hyperspectral) have shown success in KIT component detection [5,6], but these approaches are optimized for industrial contexts. To fill this gap, IoTKITs was developed to provide a comprehensive dataset of complete IoT kits captured under realistic educational settings.

However, these studies focused mainly on component-level or industrial PCB scenarios, not on the complete educational KIT recognition gap that IoTKITs aims to fill.

## 3 Data Description

This study introduces IoTKITs [7], a novel dataset targeting the holistic recognition of IoT kits commonly used in STEM education and research. IoTKITs consists of 3,200 manually annotated images spanning 32 KIT classes, including popular boards such as Arduino Uno, Arduino Nano, ESP32, Raspberry Pi, and more. Annotation was performed using the Roboflow tool with polygon-based labeling, ensuring precision in marking KIT regions. To the best of our knowledge, this is the first dataset designed specifically for comprehensive recognition of IoT educational kits. Table 1 lists the quantity of each IoT KIT included in IoTKITs dataset.

**Table 1:** List of IoT device types in the dataset.

No	Name	Quantity
1	Mega 2560 (Yellow)	100
2	Mega 2560 (Black)	100
3	Mega 2560 (Blue)	100
4	Uno (Black)	100
5	Uno (Green)	100
6	Uno Camera Shield	100
7	Uno WiFi Shield	100
8	Arduino-Due	100
9	Arduino-leonardo	100
10	Arduino-Micro	100
11	Arduino-Nano	100
12	Arduino-Pro Mini	100
13	Arduino-Zero	100
14	ESP32	100
15	ESP8266	100
16	Jetson Nano	100
17	Jetson TX2	100
18	Raspberry Pi 1 B+	100
19	Raspberry Pi 3 B+	100
20	Raspberry-Pi-1-A+	100
21	Raspberry-Pi-2-B	100
22	Raspberry-Pi-3-A+	100
23	Raspberry-Pi-3-B	100
24	Raspberry-Pi-4-B	100
25	Raspberry-Pi-5	100
26	Raspberry-Pi-Zero	100
27	Raspberry-Pi-Zero-2-W	100
28	Raspberry-Pi-Zero-W	100
29	Raspberry-Pi-Zero-WH	100
30	STM32	100
31	TelosB	100
32	Wemos	100

## 4 Experimental Design, Materials and Methods

### 4.1 Dataset collection process

To develop the dataset, a comprehensive survey was first conducted to identify the most commonly used IoT boards in teaching and research. Faculty members, researchers, and students from over 20 universities participated in the survey, which focused on gathering insights into the frequency and importance of various devices in educational and research settings. The results, summarized in Table 2, reflect the devices most frequently utilized in practical exercises and academic projects.

**Table 2:** List of universities using IoT devices in teaching.

No	Universities
1	Eastern International University
2	Lac Hong University
3	Binh Duong University
4	Ton Duc Thang University
5	University of Economics and Finance of Ho Chi Minh City
6	Vietnam Aviation Academy
7	University of Economics and Technology in Binh Duong
8	University of Transport in Ho Chi Minh City
9	University of Science and Technology in Ho Chi Minh City
10	Hanoi University of Transport
11	Ho Chi Minh City Power College
12	Thuy Loi University
13	Van Lang University
14	Saigon Van Lang College
15	Hanoi University of Transport
16	Hoa Sen University
17	International University - VNU HCM
18	International University
19	University of Information Technology
20	University of Danang - University of Science and Technology
21	University of Natural Sciences, VNU-HCM

A survey revealed 32 frequently used object classes. Consequently, the dataset was structured with a balanced 80 training and 20 testing images per class. This specific allocation enhances model training while providing diverse test data for accurate performance assessment.

Among the 3,200 images in the dataset, most are individual photographs of experimental boards, with a smaller number featuring multiple boards within the same frame. This approach aims to capture the diversity in the usage and arrangement of IoT boards in real-world educational and research environments.

**Fig. 1:**
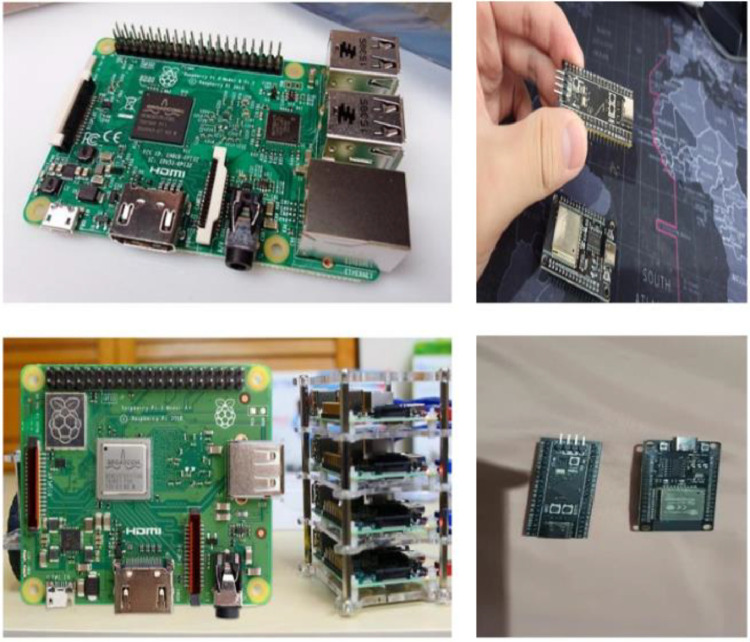
Single and multiple objects data.

**Fig. 2:**
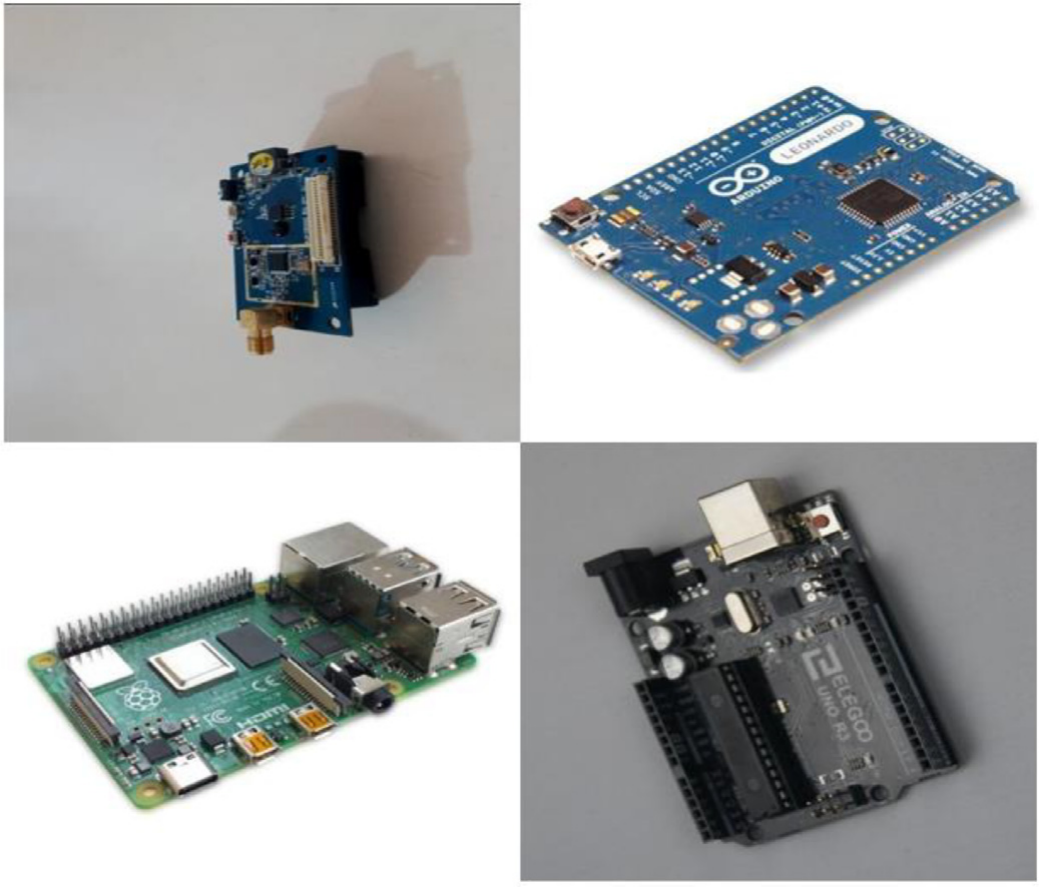
Images with a single background.

As shown in Fig. 1, Fig. 2, the dataset captures images in two distinct contexts: real-world environments and plain-colored backgrounds. Real-world images include surrounding objects, such as other devices or circuit boards, simulating practical lab or classroom conditions. Plain-colored images, mostly with white or gray backgrounds (as in Fig. 2), emphasize the main features of the boards. This approach improves object recognition across diverse scenarios, ensuring the model adapts to various lighting and contextual changes frequently encountered in real-world applications.

This approach aims to enhance the accuracy and applicability of object recognition models in practical scenarios where IoT boards are used in diverse environments. By including images with both plain and complex backgrounds, the model is expected to recognize objects accurately even under varying contexts and lighting conditions.

### 4.2 Annotation process

To improve the efficiency of model training, all images in the dataset were resized to a uniform dimension of 640 × 640 pixels. Given that the original dataset did not include annotations, manual labeling was performed using Roboflow, a widely recognized tool for its intuitive interface and cost-effectiveness. Roboflow’s “Smart Polygon Labeling” feature was utilized to generate highly precise polygonal annotations, enabling accurate delineation of object boundaries. This level of precision plays a critical role in enhancing the quality of annotations, thereby contributing to improved model performance during training and evaluation.

The annotation process involved identifying objects within each image and carefully outlining them using polygonal points. This approach was particularly effective for capturing the intricate shapes and irregular contours of IoT devices. The method ensured that even objects with complex geometries were annotated with high accuracy. In total, 3,200 images were manually labeled following this procedure.

**Fig. 3:**
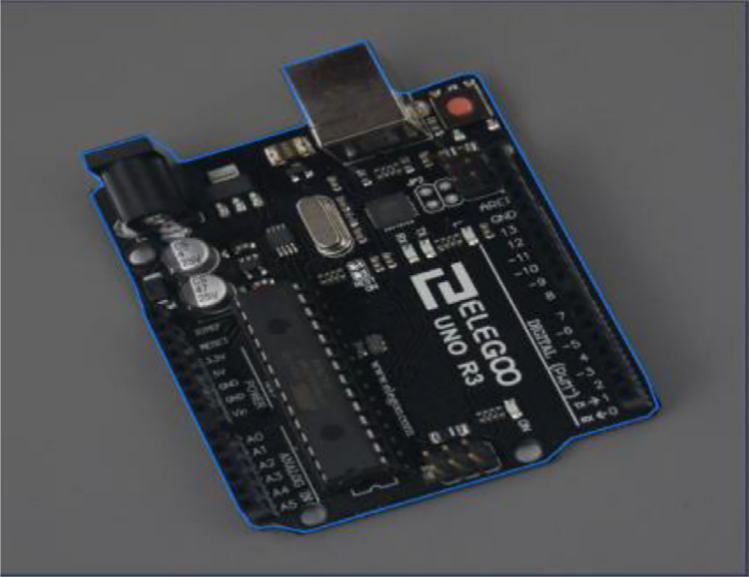
Example of IoT device labeling using Roboflow software.

Roboflow was selected as the primary annotation tool due to its user-friendly interface, flexibility, and cost-effectiveness when processing a large number of images. One of its standout features, “Smart Polygon Labeling,” allows for the automatic generation of detailed and precise polygonal labels. For example, an image of KITs labeled as shown in the Fig. 3. This feature not only improves upon traditional manual labeling methods by significantly reducing time but also ensures a high degree of accuracy in delineating object boundaries. Additionally, these polygonal labels are easily adjustable and can be refined further to enhance the quality of the training data or refined into a box shape suitable for different object detection and segmentation models.

**Fig. 4:**
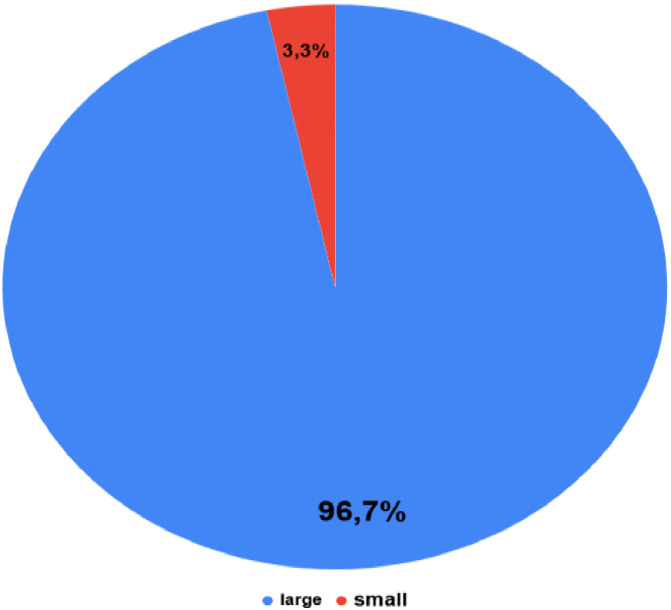
Object size distribution in images.

**Fig. 5:**
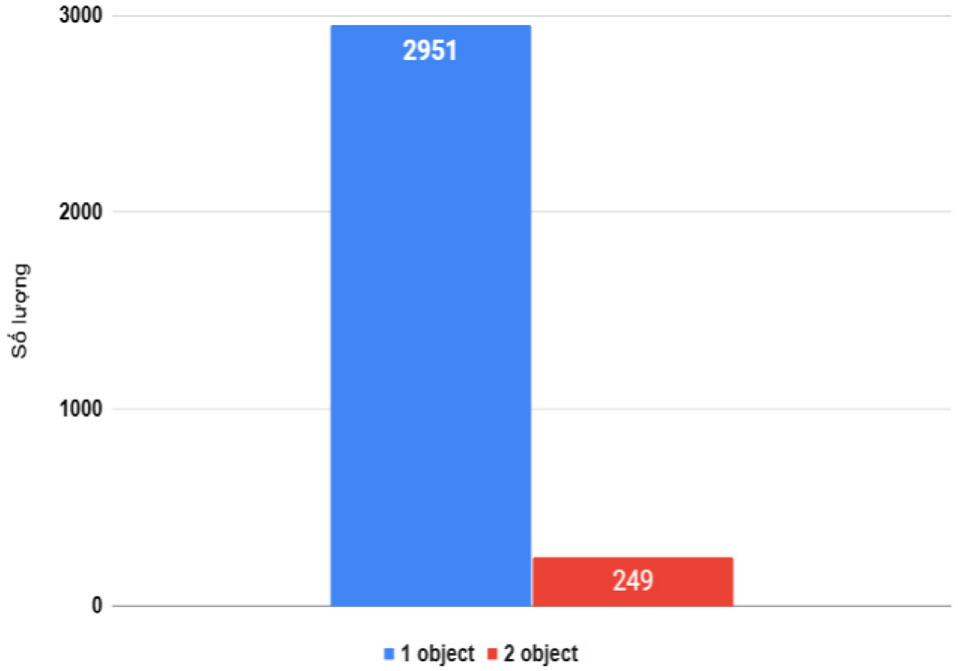
Object distribution in the dataset.

In the dataset of 3200 images, objects are classified by size as follows: objects with an area greater than 1020px are categorized as “large”, accounting for 96.7% (3095 images). On the other hand, objects with an area smaller than 224px are categorized as “small”, making up 3.3% (105 images) as in Fig. 4. This distribution indicates the dominance of “large” objects in the dataset, with only a small proportion being “small” objects. In the dataset, the images mainly contain just one KIT as shown in distribution Fig. 5.

### 4.3 Validation

To validate the utility of IoTKITs, we benchmarked four widely used object detection models: **YOLOv5, YOLOv7, Faster R-CNN**, and **SSD**. These CNN-based models were selected for their balance of detection accuracy, computational efficiency, and deployability on resource-constrained devices like Raspberry Pi and Jetson Nano.

While newer transformer-based models (e.g., DETR, Swin Transformer) have shown promise, they generally require large-scale datasets and more computational resources [8], which are less suitable for educational settings. CNN models, in contrast, are more efficient and better aligned with the goals of lightweight deployment.

### 4.4 Training setup

Each model was trained on the IoTKITs dataset using a consistent input resolution of 640 × 640 pixels. Training was run for up to 1000 epochs, with **early stopping** set at 100 epochs to avoid overfitting. Table 3 summarizes the key training parameters.

**Table 3:** The training parameters used in this research.

Model	Epochs	Batch Size	Image Size	Patience
Yolo V5	1000	32	640 × 640	100
Yolo V7				
Faster-RCNN				
SSD				

### 4.5 Evaluation metrics

Model performance was evaluated using:

**mAP@50** and **mAP@50–95**: widely used metrics for object detection accuracy

**Number of parameters (in G)**: reflects model complexity and suitability for deployment

These metrics are particularly relevant for **educational environments**, where computing resources are limited. Table 4 show the performance of 4 models.

**Table 4:** Training results of 4 models.

Stopping training early(1000)	Model	mAP 50	mAP 50-95	parameters(G)
474	Yolo v5	0.991	0.954	0.07096429
1000	Yolo v7	0.987	0.956	0.37363770
206	Faster RCNN	0.966	0.901	0.43712278
161	SSD	0.929	0.750	0.27850710

### 4.6 Results and observations

Among the tested models, **YOLOv5** achieved the best balance of performance and size, making it ideal for embedded systems used in classrooms. **YOLOv7** delivered slightly higher mAP@50–95 but required significantly more resources. **Faster R-CNN** showed strong accuracy but was the heaviest, and **SSD**, while efficient, had the lowest accuracy.

## Limitations

Although IoTKITs provides valuable annotated data for educational IoT kits, it has several limitations:

• The dataset contains only **3,200 images** and **32 classes**, which are relatively small compared to large-scale object detection datasets such as COCO or OpenImages.
• Most of the images contain **single objects** in the foreground, with only a small portion showing multiple KITs per frame, which may limit model training for multi-object detection scenarios.
• While images were captured in both plain and real-world backgrounds, **lighting variation and occlusion** are not yet extensively represented.
• The dataset has not yet been tested under **adversarial conditions** (e.g., noise, blur, occlusion) or on images captured in uncontrolled classroom environments.
• Statistical significance testing and real-hardware benchmarks (e.g., inference speed on Raspberry Pi) are planned for future work to validate model robustness and deployment feasibility.

## Ethics Statement

This work does not involve human subjects, animal experiments. Our dataset empolyed original photographs taken by the authors and kit images obtained from the following source: Kaggle repository (https://www.kaggle.com/datasets/frettapper/micropcb-images) and Roboflow Universe repository (https://universe.roboflow.com/ontheball/1a-1b), both of them are under the Creative Commons Attribution 4.0 International (CC BY 4.0) license.

## CRediT authorship contribution statement

**Thanh-Thien Nguyen:** Methodology, Software, Validation. **Anh-Tuan Nguyen Do:** Conceptualization, Software, Validation, Formal analysis, Investigation, Data curation, Writing – original draft, Visualization. **Duc-Lung Vu:** Conceptualization, Methodology, Formal analysis, Resources, Writing – review & editing, Supervision, Project administration, Funding acquisition.

## Data Availability

Mendeley DataIoTKITs (Original data)
